# Hypothalamic abnormalities and Parkinsonism associated with H1N1 influenza infection

**DOI:** 10.1186/1742-2094-7-47

**Published:** 2010-08-17

**Authors:** Alejandra González-Duarte, Lucía Magaña Zamora, Carlos Cantú Brito, Guillermo García-Ramos

**Affiliations:** 1 Department of Neurology, Instituto Nacional de Ciencias Médicas y Nutrición Salvador Zubirán, México D.F., México

## Abstract

**Objective:**

To describe a case of a young adult with severe H1N1 influenza illness associated with hypothalamic abnormalities and post-influenza parkinsonism.

**Design:**

Case report.

**Patient:**

A 22-year-old woman with H1N1 influenza infection developed encephalopathy followed by diverse hypothalamic dysfunction manifestations, sleeplessness, and persistent parkinsonian features.

**Results:**

CSF analysis, brain imaging and EEG ruled out hypoxic brain injury or other illnesses.

**Conclusions:**

A number of viruses have been associated with both acute and chronic parkinsonism. A link between parkinsonism and influenza viruses is somewhat controversial. This is the first reported case of parkinsonism following an H1N1 influenza infection.

## Background

The full incidence of neurologic complications and sequelae from the 2009 influenza A (H1N1) pandemic has yet to be determined. There is a large body of evidence that influenza can directly lead to encephalitis; however, a link with Parkinson's disease is somewhat controversial [1]. Many of the influenza viruses have not demonstrated clear neurotropism, and they rarely produce a true neuroinvasive disease. For this reason, a possible connection between postencephalic Parkinson's disease and influenza is based only on simultaneous occurrence. The recognition of neurologic post-influenzal disease has been based on coincidental occurrence with influenza pandemics, and to date there are no diagnostic serological, animal or electrographical confirmation tests. In 1974, Gamboa et al [2] found antigen material related to neurotropic influenza strain A0 in some neurons of the hypothalamus and substantia nigra in postencephalitic parkinsonian brains. Other reports have also found Influenza viral RNA in cerebrospinal fluid [3,4]. However, these findings have not been consistent. Moreover, the failure to demonstrate differences in influenza virus H1 antibodies between postencephalic Parkinson patients and idiopathic Parkinson patients, carefully matched many years after the epidemic, weighs against the persistence of influenza virus in postencephalic Parkinson patients [5] and its possible pathogenic role.

In recent years, many cases of influenza-associated acute encephalopathy have continued to emerge [5-9]. The question arises as to whether there is any evidence that points to a relationship between the virus and the concurrently neurologic emergent disease. We describe a patient with parkinsonian neurologic complications associated with severe H1N1 influenza infection.

## Case summary

A previously healthy 22-year-old Hispanic female was admitted to a local hospital after two weeks of fever of 39.5°C (103.1°F), non-productive cough, dyspnea and cyanosis. Community acquired pneumonia or influenza H1N1 was suspected, and treatment with clarithromycin, ceftriaxone and oseltamivir was initiated. She required intubation and mechanical ventilation, and was referred to our institution.

Upon her arrival, she was moderately agitated despite sedation. Her blood pressure was130/90 mmHg, with a heart rate of 100 beats per minute, a temperature of 37°C and an O_2 _saturation of 80%. Her WBC was 13,500 cel/mm^3^, with 86% neutrophils and 7.9% lymphocytes; serum glucose was 129 mg/dL, sodium 132 mEq/L, and creatinine 0.49 mg/dL. A thoracic CT scan showed bilateral infiltrates consistent with pneumonia. Ceftriaxone treatment was changed to piperazillin and tazobactam, while treatment with clarithromycin and oseltamivir was continued. She also received amantadine for 3 days, but this was discontinued after detection of H1N1 influenza RNA by RT-PCR (*CDC Realtime rTPCR, dual-labeled hydrolyisis TaqMan^® ^probes, 2009 CDC protocol for detection and characterization of swine influenza*) in a nasopharyngeal swab.

**Figure 1:**
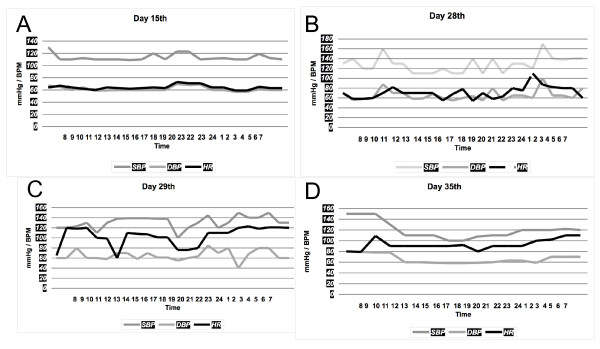
**Blood pressure and heart rate changes**. **(A) **Five days after norepinephrine infusion was stopped, the patient was stable and was weaned off the ventilator**; (B) **and **(C) **heart rate and blood pressure show random elevations, the patient was afebrile; **(D) **after initiating clonidine the vital signs were more stable. **BP**: blood pressure, **HR**: heart rate, **BPM**: beats per minute.

Over the following days, her sodium levels increased to 151 mEq/L despite treatment with free water. As the urine osmolality was persistently low, diabetes insipidus was suspected, and intranasal desmopressin was initiated. Twenty days later she was extubated. While fentanyl and midazolam were being titrated down, she became severely agitated and was treated with haloperidol. Around that time a generalized resting tremor was noted, predominately on the left leg and left arm, as well as masked face, decreased blinking, and cogwheel phenomena. She did not have elevated temperature or elevation of serum CPK levels. Haloperidol was discontinued, but the tremor did not subside. EEG and brain MRI were normal; specifically, there were no signs of pontine myelinolysis or basal ganglia abnormalities. CSF analysis was normal, with 0 cells/mm^3^, protein 39 mg/dL and glucose 47 mg/dL (serum glucose 75 mg/dL). Seven days later she developed severe autonomic cardiovascular fluctuations consistent with bouts of hypertension alternated with hypotension, brady and tachyarrhythmias. Her blood pressure ranged from 160/100 mmHg to 80/40 mmHg and her heart rate ranged from 180 beats per minute to 35 beats per minute (Figure 1). Soon after clonidine was started, her heart rate and blood pressure became stable.

Six months later she still complains of diurnal somnolence, insomnia and hyperhidrosis. She is alert and answers questions appropriately, although her behavior is disinhibited at times. Cranial nerve examination is normal. Muscle strength is 3/5 in upper and lower extremities, and there is muscle atrophy. Deep tendon reflexes are globally diminished. Plantar reflexes are flexor. She displays parkinsonian features despite the use of biperiden and clonazepam (Additional file 1).

## Discussion

We describe psychomotor agitation at onset, several hypothalamic abnormalities, sleeplessness and changes in conduct occurring in an otherwise healthy young adult with H1N1 influenza infection. The findings of neurogenic deficiency of arginine-vasopressin (AVP), autonomic hyperactivity, loss of the circadian rhythm, behavioral inappropriateness, and hyperhidrosis support the involvement of the hypothalamus. The patient still displays post-encephalic parkinsonian motor symptoms consistent of bradykinesia, tremor and "mask like" face months after the acute illness.

Paroxysmal autonomic instability has been associated with encephalitis among other intracranial catastrophes. It consists of severe blood pressure fluctuations with wide pulse pressure, tachyarrhythmias, hyperthermia and hyperhidrosis [10] with or without dystonia [11]. This dramatic response reflects direct excitation, disinhibition, or both, of sympathoexcitatory areas of the hypothalamus or the medulla [10]. Neurogenic pulmonary edema, neurocardiogenic injury, cardiac arrhythmias, and sudden death are among the most dreaded possible complications [10]. Neuroleptic malignant syndrome (NMS) is a severe idiosyncratic reaction resulting from impaired central dopaminergic transmission in the hypothalamus and basal ganglia. Two common triggers are drug-induced blockade of D2-dopaminergic receptors and sudden withdrawal of dopaminergic agonists. While the use of haloperidol and amantadine withdrawal have been linked to NMS, the lack of full recovery and the absence of CPK elevations does not support NMS in our patient.

Neurologic complications of the influenza virus are uncommon complications of a very common infection. The pathogenesis is still not fully understood. The absence of a significant inflammatory response in the CSF supports para-infectious or post-infectious immune mechanisms. The hypothalamus coordinates many complex patterns of neuroendocrine outputs, circadian rhythms, homeostatic mechanisms and behavior. Although acute hypothalamic-pituitary-adrenal axis responses to stress are generally adaptive, excessive responses can lead to deleterious effects. Specialized brain regions that lack an effective blood-brain barrier include the hypothalamus and circumventricular areas. The capillary endothelium is fenestrated to allow free passage of large proteins and other molecules. These structures are densely vascularized and may be the site of action of interleukins to elicit both fever and ACTH secretion. Several studies have proposed a central role for various inflammatory cytokines in the genesis of influenza-associated neurologic illness [9]. Rather than a direct neuroinvasion, it is possible that the hypothalamus and surrounding structures are vulnerable to the massive cytokinemia produced in patients with more severe forms of influenza. However, it is also likely that a spectrum of illnesses or underlying factors is at play, and more research needs to be done.

Sporadic cases clinically diagnosed as H1N1-associated neurologic complications attract close attention, not just as a matter of historical curiosity, but as observational evidence to obtain objective data. The severity of neurologic sequelae warrant efforts to investigate these sporadic cases to better understand various chronic neurologic conditions, especially parkinsonism.

In summary, our patient displays several hypothalamic abnormalities and post-encephalic parkinsonian features. The exclusion of other possible etiologies of acute neurologic illness, combined with supportive evidence of acute influenza virus infection, suggests that these are influenza-associated complications. Case reports like ours highlight the threat of CNS involvement. Although rare, these neurologic manifestations may be overlooked in patients with H1N1 influenza.

## Competing interests

The authors declare that they have no competing interests.

### Consent

Written informed consent was obtained from the patient for publication of this case report and accompanying images. A copy of the written consent is available for review by the Editor-in-Chief of this journal.

## Authors' contributions

AGD participated in the evaluation and care of the patient, analyzed the data and wrote the manuscript. LMG participated in acquisition of data. All authors contributed in the evaluation and care of the patient and review of the manuscript. All authors read and approved the final manuscript.

## Supplementary Material

Additional File 1 **Video 1 3GPP movie Video 1 showing persistent unilateral tremor**. The patient displays an involuntary oscillating rhythmic tremor in her left arm that is present at rest, although it is better visualized for video purposes while she is stretching her arms. A masked face is also present. These features were not present before her hospital stay. Click here for file
